# Clinical Analysis of Risk Factors of Postoperative Psychiatric Disorders in Patients With Adult Craniopharyngioma

**DOI:** 10.3389/fneur.2021.754349

**Published:** 2021-11-17

**Authors:** Rui Zhao, Pengwei Lu, Yanzhu Fan, Chuzhong Li, Chunhui Liu, Peng Zhao, Lei Cao, Hongwei Gao, Songbai Gui

**Affiliations:** ^1^Department of Neurosurgery, Beijing Tiantan Hospital, Capital Medical University, Beijing, China; ^2^Beijing Neurosurgical Institute, Capital Medical University, Beijing, China

**Keywords:** craniopharyngioma, psychiatric disorders, risk factors, hypothalamic invasion, tumor location

## Abstract

**Objective:** To analyze the risk factors relative to postoperative psychiatric disorders in adult patients with craniopharyngioma.

**Methods:** A retrospective case-control study design was used in this study. The Neuropsychiatric Inventory–Questionnaire (NPI-Q) assessment tool was used to assess psychiatric disorders in postoperative patients with craniopharyngioma at Beijing Tiantan Hospital from January 2018 to December 2020. The relationship between the psychiatric disorders and basic demographic data as well as several risk factors, such as the tumor characteristics (tumor location, tumor size, pathological finding of the tumor, etc.) and treatment-related factors (the extent of the resection), were analyzed.

**Results:** A total of 173 patients were included in this study. The prevalence of psychiatric disorders was 14.5% among adult craniopharyngioma patients. Irritability represented the most common type of psychological symptom (64%, *n* = 16), followed by agitation (36%, *n* = 9), and delusions (28%, *n* = 7). The risk factors relative to postoperative psychiatric disorders that were identified were a tumor volume larger than 7 cm^3^ (HR = 3.292, *P* = 0.042), tumor location (*P* = 0.003), hypothalamic invasion (HR = 9.766, *P* = 0.036), and gross-total resection (HR = 0.085, *P* = 0.042).

**Conclusion:** Neurocognitive assessment and intervention before and after surgery are important in patients with larger tumors, invading the third ventricle, and tumors with hypothalamic invasion. Prediction of these risk factors is essential for the treatment.

## Introduction

Craniopharyngiomas are rare, benign, and slow-growing tumors of the sellar and/or parasellar region. Due to their close anatomical proximity to the hypothalamus, the pituitary, and the optic nerves, craniopharyngiomas are frequently associated with visual, endocrine, and neurobehavioral deficits which may seriously limit functional capacity and quality of life (1, 2). Within the past three decades, advances in treatment strategies and techniques have led to decreasing mortality and less severe morbidity (3). Improved outcomes have encouraged researchers to increasingly focus attention on issues related to neurobehavioral, social, and emotional dysfunctions and quality of life, including cognitive functions (4–6).

There have been several researches focusing on pediatric patients psychiatric, cognitive, and behavioral outcomes following craniopharyngioma and pituitary adenoma surgery (7–10). Due to the different diagnostic criteria and the selection of specific populations, the incidence of postoperative psychiatric disorders in patients with craniopharyngioma is between 24 and 75% (6, 9). Following surgical intervention, the psychiatric disorders may manifest Korsakoff-like memory deficits, behaviors/personality changes, impaired emotional control, cognitive impairment, mood alteration, and psychotic symptoms (11, 12).

Previous studies have identified several risk factors of psychiatric disorders in patients with craniopharyngioma. Giese et al. (6) described a cohort of 36 patients with craniopharyngiomas; the risk factors that were found include: a tumor volume larger than 9 cm^3^, tumor extension toward/into the third ventricle or the brainstem, and resection using a bifrontal translamina terminalis or left-sided approach. A systematic review by Pascual confirms that: the histological type of the tumor, age, and postoperative treatment with chemotherapy and radiotherapy are also potentially associated with psychological deficits (13).

Psychiatric disorders are associated with the patient's reduction of quality of life, impairment in social relationships, longer rehabilitation time, poor adherence to treatment, and abnormal illness behavior (14). However, the current research is mainly focused on pediatric patients, and there is almost no research on psychiatric disorders in adult patients with craniopharyngiomas through the endoscopic endonasal approach. Therefore, in the current study, we systematically assessed the postoperative psychiatric disorders and risk factors of patients with adult craniopharyngioma.

## Patients and Methods

This study includes a total of 173 patients who underwent craniopharyngioma surgery between January 2018 and December 2020. The inclusion criteria were as follows: a histological diagnosis of craniopharyngioma, medically fit to complete the assessment, include adult cases (defined as age ≥14 years old at time of craniopharyngioma diagnosis and treatment), and surgical approaches were endonasal approach. The exclusion criteria were as follows: a history of psychiatric disorders before the diagnosis of brain tumor and a history of head trauma. This study was approved by the Ethics Committee of Beijing Tiantan Hospital, Capital Medical University. Informed consent was obtained from all participants or their legal guardians.

### Data Collection

Complete medical records were retrospectively reviewed. Patient demographic and images and details of the surgery were recorded. We recorded basic demographic information regarding age, sex, course, pathological type (adamantinomatous and squamous papillary), tumor character (calcification or not, solid or cystic), primary or recurrent surgery, tumor location (All craniopharyngiomas were simply classified into the following four subtypes based on the anatomical location for surgical approach selection: intrasellar type, intra-suprasellar type, suprasellar type, and intra-third ventricle type) (15), tumor size, hypothalamic invasion [Puget's grading system is used as the standard for hypothalamic invasion. Specifically, grade 0 (no hypothalamic damage), grade 1 (negligible hypothalamic damage or residual tumor displacing the hypothalamus), or grade 2 (significant hypothalamic damage with the floor of the third ventricle not identifiable)] (16), and extent of resection. The extent of tumor resection was confirmed by intraoperative findings and postoperative MR images acquired within 48 h after surgery. Gross-total resection (GTR) was deemed as the absence of residual tumor by these criteria, and cases in which there was any small residual tumor were classified as subtotal resection (STR).

### Psychiatric Disorders

The psychiatric disorders were assessed with the Neuropsychiatric Inventory–Questionnaire (NPI-Q), a retrospective questionnaire that measures the presence of a number of neuropsychiatric symptoms (0 = present; 1 = not present) and their severity (1 = mild; 2 = moderate; 3 = severe). It includes the items hallucinations, delusions, agitation, depression, anxiety, elation, apathy, disinhibition, irritability, motor disturbance, night-time behavior, and appetite. The NPI-Q has been accepted as a brief, reliable, informal-based assessment of neuropsychiatric symptoms assessment.

### Follow-Ups

Regular follow-ups were performed at 6 months postoperatively by outpatient, rehospitalization, or a telephone call.

### Statistical Analysis

Statistical analyses were performed using SPSS software 23.0. Univariate descriptive statistics were applied to describe the sociodemographic characteristics of study participants, as well as their tumor characteristics and treatment-related factors. For the normal distribution and equal variances data, the statistical analyses of categorical variables between two groups [the psychiatric group (cohort with craniopharyngioma with psychiatric symptoms) and non-psychiatric group (cohort with craniopharyngioma without psychiatric symptoms)] were carried out using *t-*test. A chi-square test and Fisher's exact test were used to analyze relationships or compare proportions. The correlation of risk factors and psychiatric disorders was evaluated by binary logistic regression. Statistical significance was defined as a *p* < 0.05.

## Results

### Patient Characteristics

The average age of the patients in this study was 42.03 ± 13.70 years. Of all 173 patients, 88 (50.9%) were male and 85 (49.1%) were female. In histopathological analysis, 131 (75.7%) patients showed an adamantinomatous craniopharyngioma and 42 (24.3%) a papillary craniopharyngioma. Calcification occurred in 109 (63.0%) cases. Thirty-nine (22.5%) cases were entirely solid; 61 (35.3%) cases were predominately cystic, and 73 (42.2%) cases were mixed cystic/solid. A total of 74.6% of the surgeries were primary, and 25.4% were repeat surgery for tumor recurrence. Based on preoperative MRI and CT scans, all tumors were classified into four subtypes: intrasellar type (type I, 5 cases), intra-suprasellar type (type II, 40 cases), suprasellar type (type III, 111 cases), and intra-third ventricle type (type IV, 17 cases). Tumor sizes were quantified by volumetric MRI measurements; small tumors had a volume of <7 cm^3^ (*n* = 96), and large tumors a volume more than 7 cm^3^ (*n* = 77). Hypothalamic invasion was found in 110 cases. The extent of tumor resection was divided into GTR and STR. GTR was achieved in 150 (86.7%) of the patients as confirmed by postoperative MRI (Table 1).

**Table 1:** Characteristics of the patients.

**Variables**		***N***	**%**
Age (y)	42.03 ± 13.70		
Course of disease (d)	5 (2, 12)		
Gender	Male	88	50.9
	Female	85	49.1
Pathological type	Adamantinomatous	131	75.7
	Squamous papillary	42	24.3
Calcification	No	64	37.0
	Yes	109	63.0
Tumor character	Solid	39	22.5
	Cystic	61	35.3
	Cystic and solid	73	42.2
Recurrent tumor	No	129	74.6
	Yes	44	25.4
Tumor location	Intrasellar type	5	2.9
	Suprasellar type	111	64.2
	Intra-suprasellar type	40	23.1
	Intra-third ventricle type	17	9.8
Tumor size	≤ 7 cm^3^	96	55.5
	>7 cm^3^	77	44.5
Hypothalamic invasion	No	63	36.4
	Yes	110	63.6
Tumor resection	Gross-total resection	150	86.7
	Subtotal resection	23	13.3
Psychiatric disorders	No	148	85.5
	Yes	25	14.5

Patients were divided into two groups: the psychiatric group (cohort with craniopharyngioma with psychiatric symptoms) and non-psychiatric group (cohort with craniopharyngioma without psychiatric symptoms). The psychiatric disorders rate of the group with tumor extension toward/into the third ventricle was higher than that of the group with the tumor in other locations (χ^2^ = 14.399, *P* = 0.002). In addition, the tumor size was classified as a categorized variable; the psychiatric rate increased significantly as the tumor in larger size 7.3% (≤7 cm^3^ group) vs. 23.4% (>7 cm^3^ group) (χ^2^ = 8.942, *p* = 0.003). Moreover, the psychiatric rate of the group with hypothalamic invasion was higher than that without hypothalamic invasion (χ^2^ = 8.807, *p* = 0.003; Table 2).

**Table 2:** Comparison of the two groups regarding the psychiatric disorders.

**Variables**		**No**	**Yes**	**t/χ2**	***P***
Age (y)		41.59 ± 13.93	44.60 ± 12.21	−1.015	0.312
Course of disease (d)		4.00 (2.00, 12.00)	6.00 (3.00, 10.50)		0.278
Gender	Male	77 (52.0)	11 (44.0)	0.551	0.458
	Female	71 (48.0)	14 (56.0)		
Pathological type	Adamantinomatous	113 (76.4)	18 (72.0)	0.220	0.639
	Squamous papillary	35 (23.6)	7 (28.0)		
Calcification	No	56 (37.8)	8 (32.0)	0.157	0.692
	Yes	92 (62.2)	17 (68.0)		
Tumor character	Solid	34 (23.0)	5 (20.0)	0.405	0.817
	Cystic	53 (35.8)	8 (32.0)		
	Cystic and solid	61 (41.2)	12 (48.0)		
Recurrent tumor	No	109 (73.6)	20 (80.0)	0.455	0.500
	Yes	39 (26.4)	5 (20.0)		
Tumor location	Intrasellar type	4 (2.7)	1 (4.0)	14.399	0.002
	Suprasellar type	97 (65.5)	14 (56.0)		
	Intra-suprasellar	38 (25.7)	2 (8.0)		
	Intra-third ventricle	9 (6.1)	8 (32.0)		
Tumor size	≤ 7 cm^3^	89 (60.1)	7 (28.0)	8.942	0.003
	>7 cm^3^	59 (39.9)	18 (72.0)		
Hypothalamic invasion	No	61 (41.2)	2 (8.0)	8.807	0.003
	Yes	87 (58.8)	23 (92.0)		
Tumor resection	Gross-total resection	126 (85.1)	24 (96.0)	1.349	0.245
	Subtotal resection	22 (14.9)	1 (4.0)		

### Psychiatric Disorders

The results of NPI-Q showed that 14.5% of the craniopharyngioma patients had at least one psychological symptom after operation. Table 3 shows the distribution of psychiatric disorders within the 12 major categorical axes considered among the 173 patients. A total of 12 psychiatric symptoms were reported in these series. Forty percent of patients manifested symptoms corresponding to two or three psychiatric categories. The most common symptoms were irritability (64%, *n* = 16), followed by agitation (36%, *n* = 9), and delusions (28%, *n* = 7; Table 3).

**Table 3:** Psychiatric disorders of the patients.

**Variables**	**Mild**	**Moderate**	**Severe**	**Total**
Delusions	3	1	3	7
Hallucinations	2	3	1	6
Agitation	4	2	3	9
Depression	2	2	1	5
Anxiety	1	2	2	5
Elation	2	2	0	4
Apathy	1	2	0	3
Disinhibition	0	0	1	1
Irritability	3	5	8	16
Motor disturbance	2	1	0	3
Night-time behavior	1	1	0	2
Appetite	0	1	1	2

### Neuropsychological Outcome

We tracked down the recovery of psychiatric symptoms in patients with craniopharyngioma who developed a psychiatric disorder after surgery. After a 6-month follow-up, we found that in all of the patients, the psychiatric disturbances significantly improved or disappeared, allowing them to resume their previous activities.

### Factors Associated With Psychiatric Disorders

According to univariate analysis, the patient's age, sex, as well as several risk factors such as course, pathological type, tumor character, primary or repeat surgery, tumor location, tumor size, hypothalamic invasion, and extent of resection were set as independent variables and whether a psychiatric disorder occurred was set as a dependent variable. Logistic regression analysis showed that tumor location (HR 0.577, 95% CI 0.060~0.5543, HR 0.211, 95% CI 0.015~2.869, HR 3.556, 95% CI 0.326 38.777, *p* = 0.003), tumor size (HR 3.879, 95% CI 1.526 9.861, *p* = 0.004), and hypothalamic invasion (HR 8.063, 95% CI 1.833~35.475, *p* = 0.006) were correlated with post-operative psychiatric disorder (Table 4).

**Table 4:** Factors associated with psychiatric disorders—Univariate analysis.

**Variables**		**HR**	**95% CI**	***P***
Age (y)		1.017	0.985~1.050	0.311
Course of disease (d)		0.994	0.956 1.033	0.757
Gender	Male	1		
	Female	1.380	0.588~3.239	0.459
Pathological type	Adamantinomatous	1		
	Squamous papillary	1.256	0.485~3.252	0.639
Recurrent tumor	No	1		
	Yes	0.699	0.245~1.989	0.502
Tumor size	≤ 7 cm^3^	1		
	>7 cm^3^	3.879	1.526 9.861	**0.004**
Calcification	No	1		
	Yes	1.293	0.524~3.193	0.577
Tumor character	Solid	1		
	Cystic	1.026	0.310~3.399	0.966
	Cystic and solid	1.338	0.435~4.118	0.612
Tumor resection	Gross-total resection	1		
	Subtotal resection	0.239	0.031~1.856	0.171
Hypothalamic invasion	No	1		
	Yes	8.063	1.833~35.475	**0.006**
Tumor location	Intrasellar type	1		**0.003**
	Suprasellar type	0.577	0.060~0.5543	0.634
	Intra-suprasellar type	0.211	0.015~2.869	0.242
	Intra-third ventricle type	3.556	0.326 38.777	0.298

According to multivariate analysis, tumor location (HR 0.014, 95%CI 0.000~0.488, HR 0.008, 95% CI 0.000~0.355, HR 0.089, 95% CI 0.002 3.512, *p* = 0.005), tumor size (HR 3.292, 95% CI 1.045 10.370, *p* = 0.042), hypothalamic invasion (HR 9.766, 95% CI 1.163~82.002, *p* = 0.036), and extent of resection (HR 0.085, 95% CI 0.008~0.919, *p* = 0.042) had a strong correlation with post-operative psychiatric disorder, whereas age, course, pathological type, tumor character, and primary or repeat surgery did not show correlation with post-operative psychiatric disorder (Table 5).

**Table 5:** Factors associated with psychiatric disorders -Multivariate analysis.

**Variables**		**HR**	**95% CI**	***P***
Age (y)		1.019	0.974~1.065	0.415
Course of disease (d)		0.995	0.947 1.045	0.835
Gender	Male	1		
	Female	2.036	0.733~5.658	0.173
Pathological type	Adamantinomatous	1		
	Squamous papillary	2.432	0.733~5.658	0.344
Recurrent tumor	No	1		
	Yes	0.561	0.126~2.505	0.449
Tumor size	≤ 7 cm^3^	1		
	>7 cm^3^	3.292	1.045 10.370	**0.042**
Calcification	No	1		
	Yes	2.414	0.420~13.877	0.323
Tumor character	Solid	1		0.758
	Cystic	0.778	0.159~3.812	0.756
	Cystic and solid	1.260	0.296~5.357	0.755
Tumor resection	Gross-total resection	1		
	Subtotal resection	0.085	0.008~0.919	**0.042**
Hypothalamic invasion	No	1		
	Yes	9.766	1.163~82.002	**0.036**
Tumor location	Intrasellar type	1		**0.005**
	Suprasellar type	0.014	0.000~0.488	**0.018**
	Intra-suprasellar type	0.008	0.000~0.355	**0.013**
	Intra-third ventricle type	0.089	0.002 3.512	0.197

## Discussion

There are few studies on postoperative psychiatric disorders in adult patients with craniopharyngioma, and the exact mechanism is still unclear. This study provides a detailed investigation on psychiatric disorders following surgical removal of craniopharyngiomas in adult patients. Specifically, it systematically assesses risk factors and long-term psychological outcomes. Our results showed that the incidence of postoperative psychiatric disorders in patients with craniopharyngioma is 14.5%. Patients with larger size tumor, hypothalamic invasion, and GTR had more risk of psychiatric disorder. In addition, tumor location also influenced the postoperative psychiatric symptoms.

NPI-Q is a sensitive cognitive function assessment scale, which is commonly adapted by researchers. Previous studies reported a high prevalence of postoperative psychiatric disorder, with 24–75% of patients showing neuropsychological deviations in at least one test item (6). However, our study found a relatively lower incidence than the previous reports. It is possible due to the differences in surgical approaches. There have been studies comparing the outcomes and complications between endoscopic endonasal approach and craniotomy for pediatric craniopharyngioma. Results showed that: patients in the open surgical group had a 33.3% rate of developing psychological and cognitive deficits during follow-up, while it was 18.5% in the endoscopic endonasal surgery group (17). Studies have demonstrated that endoscopic endonasal surgery can achieve a comparable or superior extent of resection over craniotomy while having a significantly lower potential of cerebrovascular injury (18).

In our research, irritability is the most common type of psychological symptom (64%, *n* = 16), followed by agitation (36%, *n* = 9) and delusions (28%, *n* = 7). This result is different from pediatric patients to some extent. Many researches have focused on the cognitive, emotional, and social behavior of children and adolescents following operations for craniopharyngioma. Children after removal of craniopharyngioma had experienced many difficulties in daily life regarding social relationships, emotion control, and learning (9). The most frequent problems in children's daily functioning included inability to control emotions, difficulties in learning, unsatisfactory peer relationships, and unattractive appearance resulting from hormonal disorders (short height and obesity). One-third of parents had problems with pathological appetite in some reports (4, 7).

**Figure 1:**
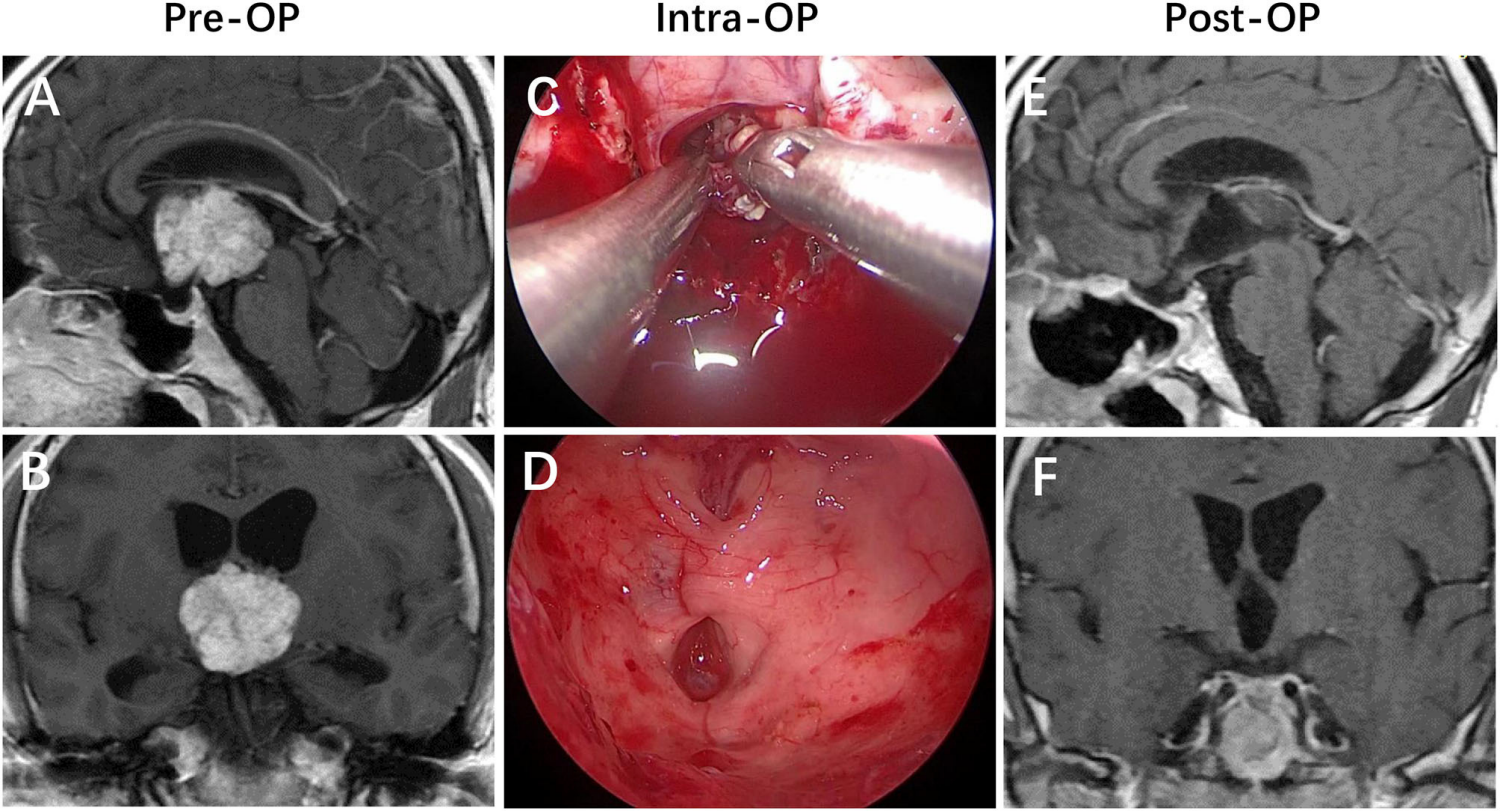
Strictly intraventricular craniopharyngioma. **(A,B)** Preoperative enhanced MRI demonstrated a mass that is localized in the third ventricle cavity with an intact floor lying below its inferior surface. **(C,D)** Intraoperative findings revealed that the patient achieved a gross-total resection of the lesion *via* the endoscopic endonasal suprachiasmatic trans-lamina terminalis corridor. **(E,F)** Postoperative enhanced MRI revealed that the lesion was totally removed, with the posterior wall of the third ventricle preserved intactly.

In our study, tumor volume is an independent risk factor for postoperative psychiatric disorder. The psychiatric disorder rate was 23.4% in the group who had tumor volume >7 cm^3^, while 7.3% in tumor volume ≤7 cm^3^ (χ^2^ = 8.942, p = 0.003). This result is consistent with the study by Giese et al. (6). The reason may be that larger tumor volume is more aggressive to the surrounding brain tissue, which causes severe damage to the function of normal structures. Tumor location or extension in/toward the third ventricle is the risk factor for postoperative mental disorders, which are consistent with the results of previous studies (6, 13). A greater frequency of hypothalamic dysfunction has been reported for craniopharyngiomas invading the third ventricle (Figure 1) (19, 20).

**Figure 2:**
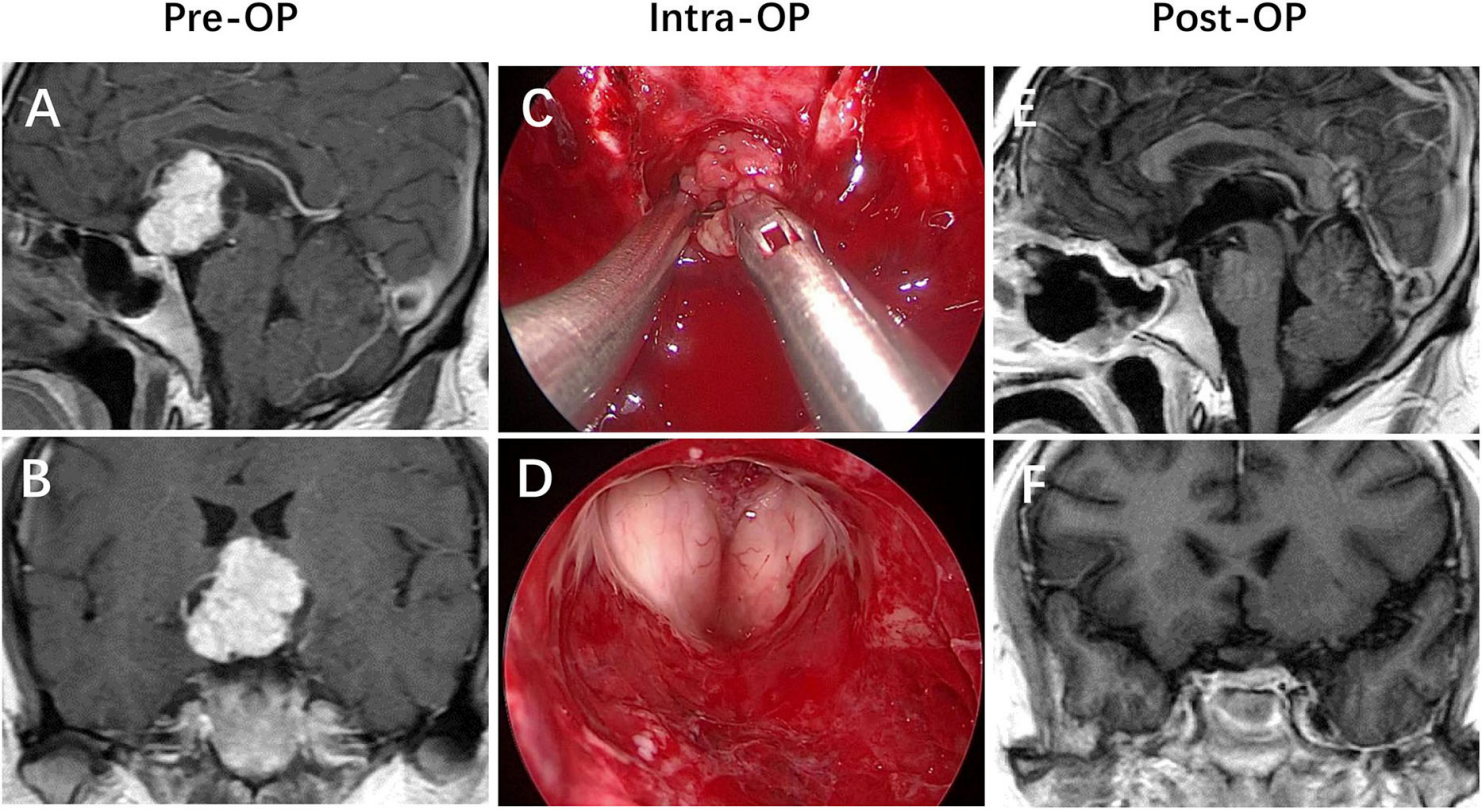
Not strictly intraventricular craniopharyngioma. **(A,B)** Preoperative enhanced MRI demonstrated that a mass had primarily developed within the neural tissue of the third ventricle floor (TVF), progressively replacing part of the floor while expanding into the third ventricle cavity, in which expansion of the tumor has breached the TVF. **(C,D)** Intraoperative views revealed that the patient achieved a gross-total resection of the lesion *via* the endonasal endoscopic endonasal infrachiasmatic corridor. **(E,F)** Postoperative enhanced MRI revealed that the lesion was totally removed, with the posterior wall of the third ventricle and the pituitary stalk preserved intactly.

**Figure 3:**
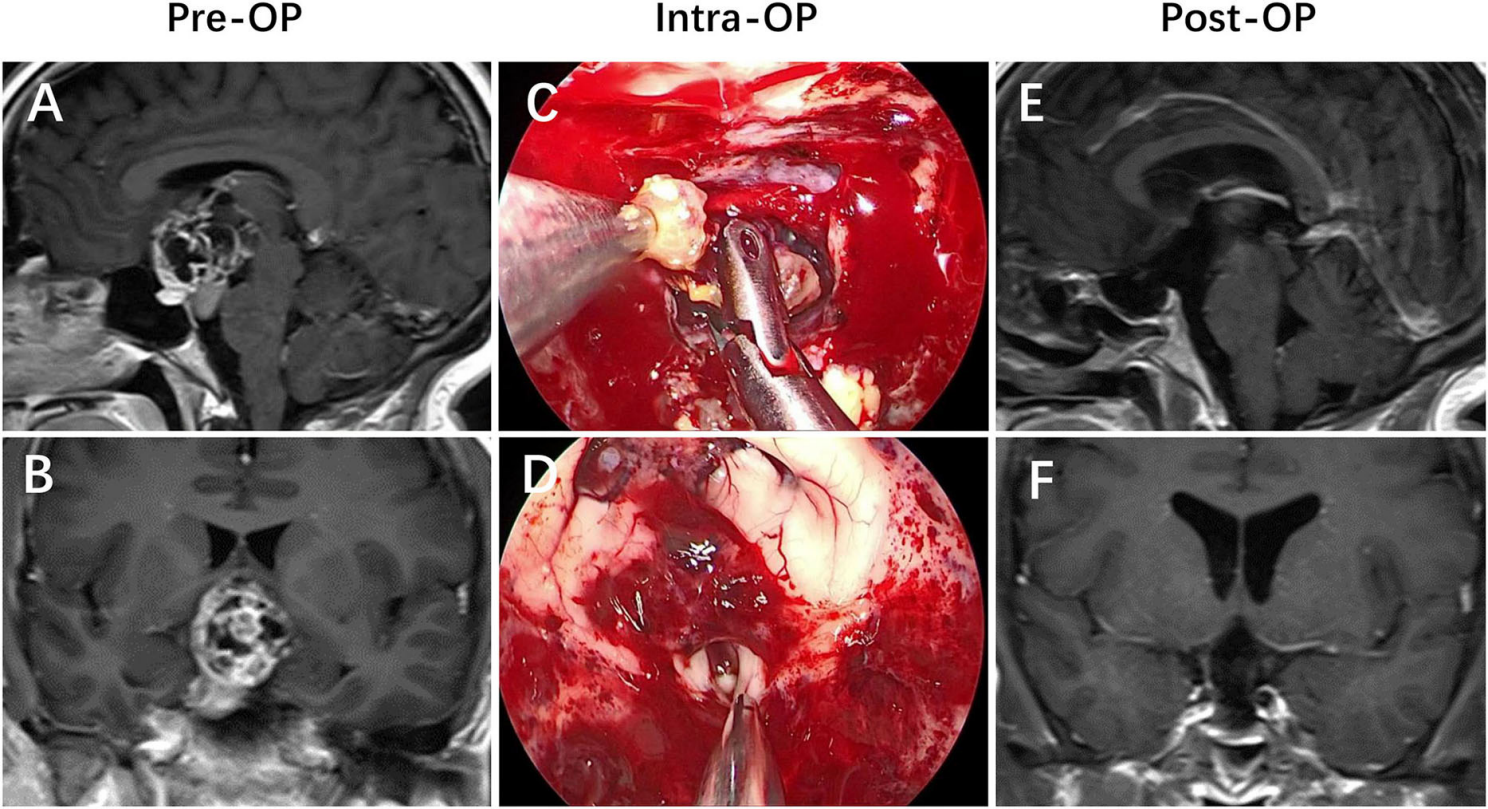
Craniopharyngioma with hypothalamic invasion. **(A,B)** Preoperative enhanced MRI demonstrated that an extraintraventricular mass developed from the pituitary gland and/or the pituitary stalk that had invaded the third ventricle after breaking through the third ventricle floor. **(C,D)** Intraoperative views revealed that the patient achieved a gross-total resection of the lesion *via* the endonasal endoscopic endonasal infrachiasmatic corridor. **(E,F)** Postoperative enhanced MRI revealed that the lesion was totally removed, with the TVF opened.

**Figure 4:**
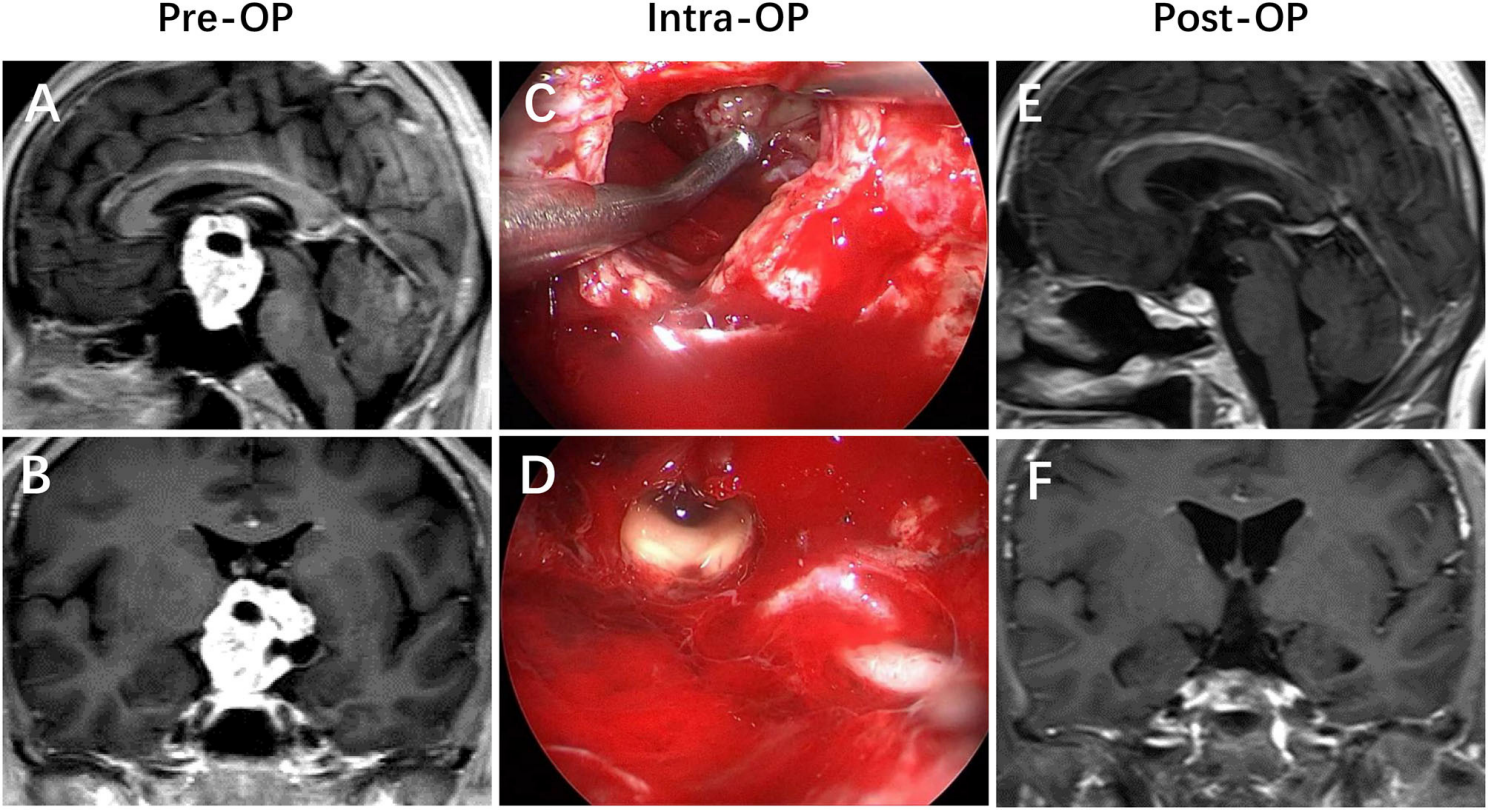
Craniopharyngioma with a large volume. **(A,B)** Preoperative enhanced MRI demonstrated that a giant suprasellar mass developed from the pituitary stalk that pushed the TVF upward, mimicking an intraventricular position. **(C,D)** Intraoperative views revealed that the patient achieved a gross-total resection of the lesion *via* the endonasal endoscopic endonasal infrachiasmatic corridor. **(E,F)** Postoperative enhanced MRI revealed that the lesion was totally removed, with the TVF opened.

Hypothalamic invasion is considered to be an independent risk factor in our study. The exact mechanism of psychiatric disorder is still unclear; however, hypothalamus injury is a relatively recognized reason (21). The central position of the hypothalamus around the third ventricle serves as the convergence point of numerous neural pathways connecting different brain areas. This diencephalic region hosts the headquarters of the brain circuitry involved in monitoring the continuous changes of the internal medium as well as external conditions to coordinate the appropriate neuroendocrine responses and active behaviors to restore body homeostasis and mental balance. Therefore, any lesion invades into the hypothalamic centers that participate in the integration of emotional and behavioral responses, which would cause potentially structural and functional damage, resulting in the development of numerous psychiatric disorders (13, 21–23). This mechanism may explain why the rate of psychiatric disorders in the group of patients with hypothalamic invasion was higher than that without hypothalamic invasion (χ^2^ = 8.807, *p* = 0.003) (Figures 2–4).

Multivariate logistic regression analysis showed that total tumor resection is the risk factor for the psychological disorders. The choice of optimal treatment strategies for craniopharyngioma remains a controversial subject (15). Because of the local recurrence that has highly occurred after partial surgical resection, total resection is advocated by many authors. But other reports also revealed that radical surgery and irradiation can result in severe damage to the optic pathway and hypothalamic–pituitary axis, thus, decreasing the prognosis of postoperative psychological outcomes. Previous studies also have found that total tumor resection affects patients' long-term quality of life (24). Based on these reports, when selecting the best treatment strategies for patients with craniopharyngiomas, it is important for surgeons to consider not only the tumor resection extents and surgical treatment outcomes but also the functional and psychological complications or long-term quality of life of the patients.

Nursing staff and a multidisciplinary team (MDT) should take the individualized and professional care of patients with mental disorders from the following aspects. Firstly, the neurosurgeons work closely with the nursing staff to identify early enough the cognitive impairment of the patient through daily observation or testing. Secondly, endocrinologists and clinical neuropsychologists should be involved in the perioperative management of patients as soon as possible. Thirdly, it is important to create a safe, comfortable treatment environment for the patients. In addition. different nursing and communication skills should be adopted for patients with different mental disorders. For irritable patients, nursing staff should avoid verbal and behavioral stimulations to these patients. And for patients with delusions and hallucinations, even if the patient's description of certain situations is not in line with the reality, nurses should be good at listening, expressing belief, and facilitating the establishment of a good nursing relationship. Furthermore, proper restraint can ensure the safety of patients; however, improper measures to restrict the activities of patients will result in obviously rebellious psychology, such as increased restlessness, loss of dignity, fear, and other changes. Therefore, when the patient's condition improves, nurses should remove the restraints on the patients in time. Finally, moderate psychotropic drugs were used for patients with severe cognitive dysfunction.

The treatment and care for craniopharyngioma is multidisciplinary, involving the cooperation of oncological surgery, medical oncology, radiotherapy, chemotherapy as well as psycho-social support and rehabilitation and, when cancer is not treatable, palliative care (14). The management of psychological disorders also relies heavily on the decision-making process in MDT, composed of dedicated experienced neurosurgery specialists and neuropsychologists, assisted by specialists from adjoining branches. A mutual exchange of specialist opinions in such fields can reach a more satisfactory treatment strategy in accordance with the scientific community standards.

## Conclusion

This exploratory study is a first experimental study toward the identification of factors predicting psychological disorders after craniopharyngioma resection in adult patients in our center. The results of this study may provide the surgeon a reference to optimize the treatment plan of craniopharyngioma while maintaining a functional and psychological balance. Recommendations for assessment and intervention to psychiatric and psychosocial disorders across the trajectory of cancer are therefore considered essential in every cancer center, institute, hospital, and community service, in order to warrant the reasonable psychological outcomes and qualities of life for craniopharyngioma patients. Furthermore, our study may also provide a unique opportunity to further our understanding on the role of hypothalamus in the integration of emotional and behavioral information.

## Data Availability Statement

The original contributions presented in the study are included in the article/supplementary material, further inquiries can be directed to the corresponding author.

## Author Contributions

RZ and PL: analyzed the data and drafted the manuscript for intellectual content. SG: surgery treatment, interpreted the data, and revised the manuscript for intellectual content. HG: patients management and follow-up and design and conceptualized study. LC: surgery treatment and revised the manuscript for intellectual content. PZ, CLiu, and CLi: surgery treatment and patients management and follow-up. YF: design and conceptualized study. All authors contributed to the article and approved the submitted version.

## Funding

This study was supported by the Beijing Municipal Science & Technology Commission (Z19110700660000) and Beijing Hospitals Authority Clinical Medicine Development of Special Funding Support (XMLX202108). The authors have no personal financial or institutional interest in any of the drugs, materials, or devices described in this article.

## Conflict of Interest

The authors declare that the research was conducted in the absence of any commercial or financial relationships that could be construed as a potential conflict of interest.

## Publisher's Note

All claims expressed in this article are solely those of the authors and do not necessarily represent those of their affiliated organizations, or those of the publisher, the editors and the reviewers. Any product that may be evaluated in this article, or claim that may be made by its manufacturer, is not guaranteed or endorsed by the publisher.
